# Strip grazing stockpiled annual forages: impact on carrying capacity and cattle performance

**DOI:** 10.1093/tas/txae159

**Published:** 2024-11-19

**Authors:** Shelby L Davies-Jenkins, Devin A Jakub, Abigail M Sartin, Zac E Carlson, Mary E Drewnoski

**Affiliations:** Department of Animal Science, University of Nebraska, Lincoln, NE 68583, USA; Department of Animal Science, University of Nebraska, Lincoln, NE 68583, USA; Department of Animal Science, University of Nebraska, Lincoln, NE 68583, USA; Department of Animal Science, University of Nebraska, Lincoln, NE 68583, USA; Department of Animal Science, University of Nebraska, Lincoln, NE 68583, USA

**Keywords:** annual forage, beef cattle, cover crop, grazing management, harvest efficiency

## Abstract

Strip grazing can increase forage utilization, though it has been shown to decrease individual animal performance. The objective of this study was to evaluate forage utilization and cattle performance when strip grazing (**STRIP**) vs. continuously grazing (**CONT**) stockpiled annual forages. Experiment 1 consisted of a mix of Jerry oats (*Avena sativa*) and Trophy rapeseed (*Brassica napus*) while experiment 2 was a 17-species mix in which the forage mass was predominantly pearl millet (*Pennisetum glaucum*), German millet (*Setaria italica*), and browntop millet (*Urochloa ramosa*). Fields were divided into six 6.3-ha (experiment 1) or 4.1-ha (experiment 2) paddocks which were blocked by location. Treatment was randomly assigned within block (*n* = 3 replicates per treatment per experiment). Grazing was initiated in November and terminated in February. This resulted in a total of 83 and 54 grazing days for experiments 1 and 2, respectively. Forage was allocated to STRIP twice a week. The STRIP steers gained 16% less (*P *= 0.01) per day in experiment 1, but in experiment 2, there was no difference (*P *= 0.56) between treatments. Compared to CONT, the carrying capacity (AUM/ha) of STRIP was increased (*P *= 0.03) by 81% in experiment 1 and tended to increase (*P* = 0.10) in experiment 2. Gain per hectare increased (*P* = 0.02) for STRIP by 56% in experiment 1 and by 31% in experiment 2. Strip grazing stockpiled annual forages can be an effective way to increase carrying capacity and gain per hectare during the fall and winter months but effects on individual animal performance are inconsistent. Disparities in response to strip grazing could stem from differences in forage quality and allocation. Further research is needed to refine and optimize the utilization of this management approach.

## Teaser

This study evaluated the effectiveness of strip grazing stockpiled annual forages, revealing its potential to increase carrying capacity and gain per hectare during fall and winter.

## INTRODUCTION

Grazing dormant forages or crop residues has previously been found to be an effective way to reduce winter feed costs compared to feeding a total mixed ration or hay in a drylot. Kelln et al. (2011) have found that extensive feeding systems had an 18% lower cost compared to a drylot system. However, in the last 20 years across the United States, pasture rental rates have increased by an estimated 67% (USDA NASS, 2023) and pasture availability has declined as many historic pastures have been converted to cropland (Lark et al., 2015).

Despite these challenges, there is an opportunity to leverage the land used for small cereal grain cultivation, which amounted to just over 31 million hectares in the United States in 2021 (USDA NASS, 2021). Following the summer harvest of small cereal grains, there remains a substantial window for capturing growing degree days through photosynthesizing plant material during an otherwise fallow period. By planting annual forage crops on this land, producers can increase diversification of the cropping system (Faé et al., 2009), access an alternative winter forage source for livestock (Deen et al., 2019), and increase soil health (Fageria et al., 2005).

Though these hectares offer much potential, due to the seasonality of their availability, grazing management is a key component that impacts the overall utilization and therefore profitability of the annual forage. Strip grazing can increase forage utilization by allocating animals to a smaller portion of a larger paddock for relatively short times without the use of a back fence (Allen et al., 2011). Though strip grazing has been shown to effectively increase the productivity of actively growing pastures (Blount et al., 1991), its primary usage is for stockpiled forages. Forages are stockpiled when they are allowed to accumulate during times of active growth for later grazing, often when the forage is not actively growing or growing very slowly (Allen et al., 2011). Generally, strip grazing is thought to result in greater harvest efficiency in comparison to continuous grazing, thus allowing more animal grazing days from the same land. This usually comes at the cost of reduced forage selectivity and thus reduced individual performance, such as reduced average daily gain (**ADG**) for growing calves (Blount et al., 1991). Ultimately, this leads to the question, can producers achieve an overall higher level of animal productivity of the herd on their current land base by utilizing strip grazing.

To date, scant research has directly compared continuous grazing to strip grazing, particularly within the context of stockpiled annual forages. Consequently, this study aims to address this knowledge gap through 2 on-farm experiments conducted in Nebraska, evaluating the effects of strip grazing vs. continuous grazing on cattle performance when utilizing various annual forage resources during the late fall and winter.

## MATERIALS AND METHODS

All procedures used were reviewed and approved by the University of Nebraska—Lincoln Institutional Animal Care and Use Committee (IACUC #1980 and 1785).

### Experiment 1—Eastern Nebraska Year 1: Oats and Brassicas

A 37.6-ha irrigated field near Mead, NE (41.215193, −96.469218) was used. The soil was Yutan silty clay loam (52%), Tomek silt loam (18%), and Filbert silt loam (14%) with 0% to 6% slope (Web Soil Survey, n.d.). Thus, the majority of the soil would be classified as Alfisol (Yutan) and the remaining as Mollisol (Tomeck and Filbert). Following spring, oats harvest was planted using a no-till drill with 56 kg/ha of Jerry oats (*Avena sativa*) and 3.4 kg/ha of Trophy rapeseed (*Brassica napus*). The drill was set for a 20.3 cm row spacing and planted occurred on August 12, 2020. Post emergence, nitrogen was applied at a rate of 42 kg N/ha. The field received 16.5 ha-mm of water through a pivot while the cover crop was actively growing. The field was divided into 6 paddocks (6.3 ha each) for a total of 3 replicates per treatment by a single-strand electric wire fence. Treatments were strip grazing (**STRIP**) vs. continuous grazing (**CONT**). The 6 paddocks were separated into 3 blocks of 2 paddocks with 2 blocks containing only irrigated land and 1 block including dryland corners.

Steers (*n* = 84) were stratified by initial body weight (**BW**; 238 ± 17 kg) and assigned randomly to 1 of 6 groups (*n* = 14 per group). Each group was then assigned randomly to a paddock. This resulted in a stocking rate of approximately 2 steers per hectare. Seven of the 14 steers in each group were randomly designated as testers and used to measure animal performance, as forage was expected to become limiting in CONT paddocks before STRIP paddocks. The other 7 steers from each paddock were used to graze any remaining forage in the STRIP paddocks after the trial was completed. Prior to weighing to equalize gut fill, steers were limit fed 2% of the BW a diet consisting of 50% Sweet Bran (Cargill Wet Milling, Blaire, NE), and 50% alfalfa hay (dry matter [**DM**] basis) for 5 d (Watson et al., 2013). To establish initial BW steers were then weighed for 2 consecutive days (days 0 and 1) using a hydraulic squeeze chute with load cells mounted on the chute (Silencer, Moly Manufacturing Inc., Lorraine, KS: scale readability ± 0.90 kg). Steers were implanted with 36 mg of zeranol (Ralgro, Merck Animal Health, De Soto, KS) on day 1 during the initial weighing process. Grazing began on November 12, 2020 and was terminated on February 3, 2021 (83 d) when the average forage height of CONT was 5.1 cm, which was considered limiting. The STRIP treatment groups were given access to new forage approximately twice a week. The area allocated (0.162 ha; SD ± 0.0811 ha per move) and timing of allocation (every 3.86 d; SD ± 1.3 d) for STRIP was adjusted based on visual appraisal to achieve the target post-graze height of 5.1 cm. The goal was to have similar post-graze forage mass for CONT and the grazed STRIP areas at the end of the grazing season. The STRIP cattle were not backfenced. Steers grazing continuously had access to 6.3 ± 0.00 ha (0.45 ha/steer) while STRIP calves used a total of 3.4 ± 0.55 ha (0.25 ha/steer). Following grazing termination, tester cattle were limit fed at 2% of their BW for 7 d and then weighed for 3 consecutive days with weighing procedures being the same as those used for initial BW (Watson et al., 2013). A free choice mineral supplement (containing calcium, salt, zinc, manganese, copper, cobalt, iodine, and selenium) was provided ad libitum to steers in each paddock during the grazing period. To estimate carrying capacity, animal unit month (AUM) was calculated as follows: the number of animal units (14 head per group multiplied by average BW divided by 454 kg) multiplied by the total number of grazing days (83 d) was divided by the number of hectares that were utilized during the grazing season for each treatment paddock, resulting in animal units/ha. Then this value was divided by the average number of days in a month (30.5), resulting in AUM/ha.

Prior to grazing (day −14; October 29, 2020), forage was clipped at ground level and sorted by species to determine forage mass. Four locations (0.91 × 0.61 m) in each of the irrigated paddocks and 5 locations in each of the paddocks containing the dryland corners were clipped (3 from the irrigated and 2 in the dryland portion of the paddock). On days 22 (December 3, 2020), 42 (December 23, 2023), and 71 (January 21, 2021) of grazing, biomass was clipped again from 4 random locations in each of the CONT paddocks. On these days for STRIP, 2 non-grazed locations were clipped in the strip that would be allocated next. No final biomass clippings were able to be collected, as steers from an adjacent field grazed in the experimental paddocks prior to sample collection. Quality samples of oats and rapeseed were collected on the same days as forage mass and were cut at ground level, either at random within the entire paddock for CONT or randomly within the entire ungrazed paddock portion for STRIP.

Forage samples dried for 48 to 72 h in a 60°C forced air oven. Quality samples were further analyzed by species for DM, organic matter (**OM**), crude protein (**CP**), and digestible organic matter (**DOM**) to determine nutritive value. Quality samples were first ground to pass a 1-mm screen using a Wiley Mill (Thomas Scientific, Swedesboro, NJ). Sub-samples were then placed in a forced air oven for 24 h at 105°C to determine DM and then subsequently placed in a muffle furnace for 6 h at 600°C (AOAC, 1999) to determine OM. Analysis of CP was conducted using the combustion method (AOAC, 2006) by Ward Laboratories (Ward Laboratories, Inc., Kearney, NE). The DOM was calculated by determining the in vitro OM digestibility (IVOMD) using methods described by Tilley and Terry (1963). Briefly, this consisted of incubating samples in buffered rumen fluid for 48 h. A modification to their methods was made by adding urea to McDougall’s buffer (McDougall, 1948) at a rate of 1 g urea/L buffer solution, to ensure that rumen fluid microbes would have adequate nitrogen availability (Weiss et al., 1992). The inclusion of blanks was used in each in vitro run and to adjust for any feed particle contamination from the inoculum. Following incubation, samples were filtered through Whatman 541 filter paper (22 µm pore size), dried at 60°C for 24 h, then placed in crucibles and heated in a muffle furnace at 600°C for 6 h. A total of 2 runs (each containing all samples) were conducted with the inclusion of 5 hay standards to which in vivo (total tract) digestibility (51% to 60% range) was known and were used to adjust IVOMD values to be similar to in vivo by adding an additional 6.5 and 6.6 percentage units for run 1 and 2, respectively. The resulting IVOMD was then multiplied by the OM content of the sample to express forage digestibility as DOM. This value (DOM) serves as an evaluation of the energy content of the forage and is a proxy for total digestible nutrients.

### Experiment 2—Eastern Nebraska Year 2: Diverse Annual Mix

A 24.3-ha irrigated field near Mead, NE (41.205611, −96.516107) was used. The soil was Filbert silt loam (36%), Tomek silt loam (27%), and Yutan silty clay loam (23%), with 0% to 6% slope (Web Soil Survey, n.d.). Following wheat harvest in mid-July, a 17-species mix which included warm- and cool-season grasses, legumes, and forbs was planted at a rate of 56 kg/ha. In total, 4.5 kg/ha Pearl millet (*Pennisetum glaucum*), 2.2 kg/ha German millet (*Setaria italica*), 1.1 kg/ha browntop millet (*Urochloa ramosa*), 11.2 kg/ha winter triticale (×*Triticosecale* Wittmack), 9.0 kg/ha spring oat (*A. sativa* L.), 2.2 kg/ha mung beans (*Vigna radiata* L.), 3.4 kg/ha cowpeas (*Vigna* Savi), 4.5 kg spring pea (*Pisum sativum* L.), 3.4 kg/ha hairy vetch (*Vicia villosa* Roth), 1.1 kg/ha wollypod vetch (*Vic. villosa* Roth *ssp. Varia*), 2.2 kg/ha chickling vetch (*Lathyrus sativus* L.), 1.1 kg/ha balansa clover (*Trifolium michelianum* Savi ssp. *Balansae* (Boiss.) Ponert), 1.1 kg/ha sunflower (*Helianthus annuus* L.), 5.6 kg/ha buckwheat (*Fagopyrum esculentum* Moench), 1.7 kg/ha flax (*Linum usitatissimum* L.), 1.1 kg/ha dwarf Essex rapeseed (*B. napus* L.), and 0.6 kg/ha turnip (*B. rapa*) were planted. No irrigation or nitrogen fertilizer was applied. Similar to experiment 1, treatments were arranged in a completely randomized block design and the experimental unit was paddock. The 3 blocks utilized and were arranged by location in the field. Treatment (CONT or STRIP) was randomly assigned to one of the two 4.0-ha paddocks per block for a total of 3 replicates per treatment. Twice weekly (4.15 ± 1.82 d) the STRIP groups were allocated new forage strips when approximately 40% of the forage (based on visual estimation) in the previous strip had disappeared. The area for the next strip was determined based on utilization of the previous strip with a target of allocating 3 to 4 days’ worth of forage at 40% utilization. The goal was to have similar post-graze biomass (40% disappearance of initial mass) for CONT and the grazed STRIP areas at the end of the grazing season. The mean area for the strips across the grazing season was 0.176 ± 0.0287 ha.

Steers (*n* = 60) were stratified by initial BW (288 ± 0.73 kg) with 10 steers assigned to each paddock. All weighing and animal care procedures were the same as described for experiment 1. Grazing was initiated on December 9, 2021 and terminated on February 1, 2022 (54 d) when the average forage disappearance of the CONT was approximately 40% of the initial forage mass. Steers grazing continuously had access to 4.04 ± 0.20 ha (0.40 ± 0.02 ha/steer) while STRIP calves used 2.89 ± 0.20 ha (0.29 ± 0.02 ha/steer). Carrying capacity was calculated using the same method as in experiment 1.

In both STRIP and CONT, 5 locations per paddock for mass and nutritive value were clipped prior to grazing initiation (pre-graze) and 5 clippings for mass analysis were collected following grazing termination (post-graze). Due to a laboratory error, no forage quality data for post-graze measurements were able to be analyzed. To collect forage measurements, each paddock was divided into 5 zones of equal area (0.32 ha) and forage mass was clipped from a random location (0.91 × 0.61 m) at ground level within each of the 5 zones. However, for post-graze, no samples were taken in the ungrazed area of the STRIP paddocks (i.e., zone 5 was not sampled). Pre-graze clippings were collected on December 3, 2021, and post-graze clippings were collected on February 7, 2022. Given the temperature, no forage growth would be expected during the grazing period. Disappearance was estimated by dividing the carrying capacity in AUM/ha by the amount of forage that disappeared on a hectare basis throughout the grazing season (pre-graze mass minus post-graze mass). The resulting number is reported as kilogram of DM disappearance per AUM.

Following collection, forage samples were sorted by plant functional type (grasses, grass seedheads, legumes, forbs, and sunflower heads) and pre-graze samples were analyzed for the nutritive content including DM, OM, CP, and DOM. Additionally, the grass seedheads were sent to Dairyland Laboratories (Arcadia, WI) for analysis of starch (method as described by Vidal et al., 2009) and the sunflower seedheads were sent for ether extract analysis (AOAC Official Method 920.39).

### Economic Analysis

To evaluate the economics of using the 2 grazing treatments, the establishment cost of the forage mix and costs associated with grazing were calculated (Table 1). All but irrigation and temporary perimeter fencing costs were the actual costs charged. Irrigation cost per hectare was calculated using the rates from the 2020 Nebraska Crop Budgets and the metered amount of water applied (Klein et al., 2019). Fencing costs were assumed to be a temporary single-wire electric fence. Labor was charged at $20/h (McClure and Jansen, 2020). The labor involved with health checks and hauling water was applied equally to both treatments. Steers were assumed to drink 20 L/steer of water daily (NASEM, 2016). Three water tanks (2,650 L) were used and shared between adjacent paddocks, resulting in the estimated need to refill water tanks every 4.5 d (approximately twice weekly). The time to fill tanks was estimated at 2 h for a total of 4 h/wk. The additional labor required for moving the STRIP fence twice weekly was estimated at 0.5 h per move per group. This was then multiplied by the number of weeks grazed and divided by the number of hectares grazed for an additional labor cost for STRIP.

**Table 1.** Partial budget used in cost of gain calculation for steers grazing continuously (**CONT**) or by strip grazing (**STRIP**)

Estimated costs, $/ha	CONT		STRIP	
	Experiment 1	Experiment 2	Experiment 1	Experiment 2
Seed cost	$26.69	$123.55	$26.69	$123.55
Seeding cost	$29.65	$29.65	$29.65	$29.65
Fertilizer^1^	$37.07	—	$37.07	—
Fertilizer application	$21.62	—	$21.62	—
Irrigation^2^	$37.11	—	$37.11	—
Pre-plant herbicide	$46.38	—	$46.38	—
Herbicide application	$17.30	—	$17.30	—
Fence	$12.36	$12.36	$12.36	$12.36
Water & health checks	$22.24	$22.24	$22.24	$22.24
Labor^3^	—	—	$69.29	$44.87
$/ha	$250.42	$187.80	$319.71	$232.67

^1^Nitrogen applied at a rate of 42 kg/ha of actual N.

^2^Total water applied = 16.5 ha-mm.

^3^Calculation factors: $20/h, fence moved twice weekly, 30 min for each fence move = 1 h/wk.

### Statistical Analysis

All data were analyzed using the Mixed procedure of SAS (SAS Institute, Inc., Cary, NC) as a complete randomized block design with location in the field being the blocking factor. Paddock was considered the experimental unit for all analysis. Treatment and block were used as fixed effects in the models for steer BW, ADG, carrying capacity, gain per hectare, and cost of gain. For experiment 1, forage nutritive value was analyzed as a repeated measure with paddock within block included in a random residual statement and paddock within block as the subject. The model contained treatment, sampling date, and interactions as fixed effects. Nutritive value in experiment 2 was not statistically analyzed due to a limited amount of sample material available from 3 of the 5 functional groups. Treatment means were separated using the pdiff statement when the *F*-test was significant. Treatment differences were considered significant when *P* ≤ 0.05 and considered a tendency between *P* > 0.05 and *P* ≤ 0.10.

## RESULTS

### Experiment 1—Eastern Nebraska Year 1: Oats and Brassicas

**Figure 1.**
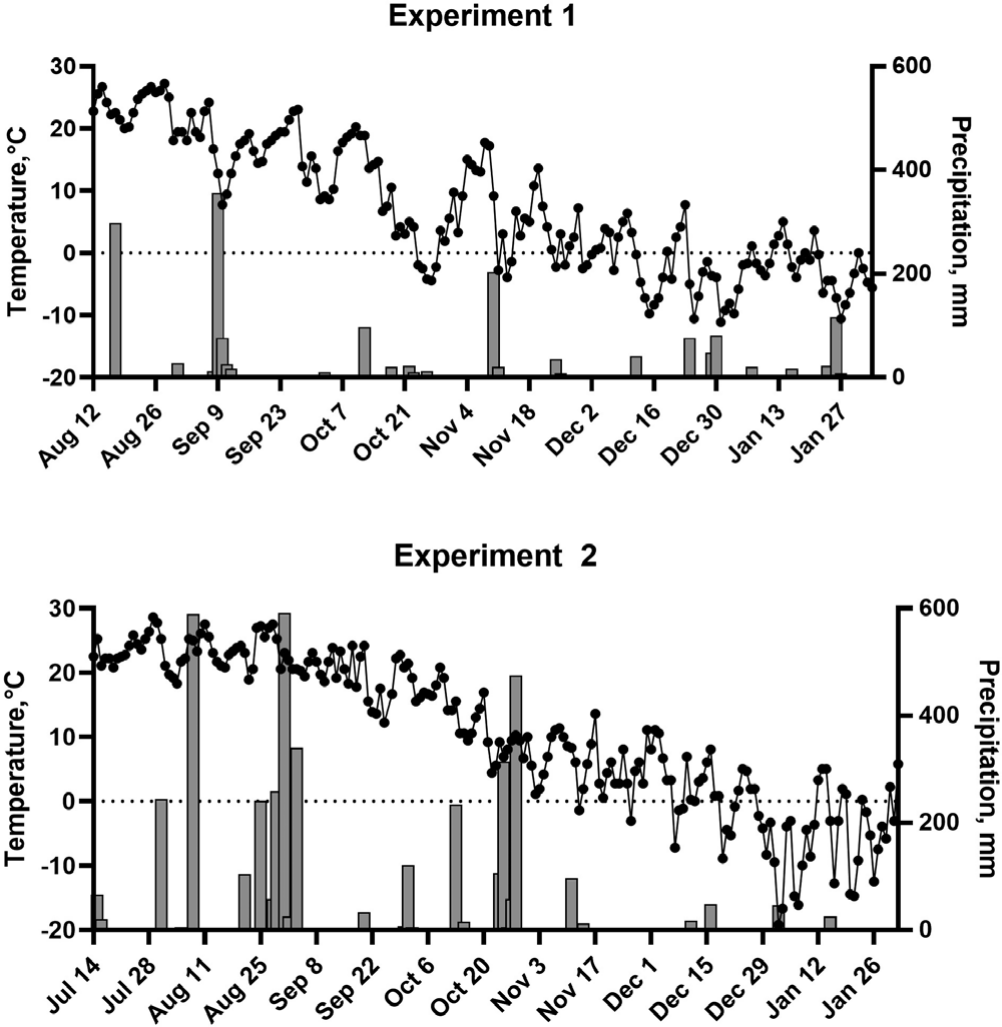
Daily precipitation (bars) and average temperature (dots) over the growing and grazing seasons. In experiment 1, an oat-rapeseed mix was planted on August 12, 2020 and steers began grazing on November 12, 2020. In experiment 2, a diverse annual forage mix dominated by warm-season grasses was planted on July 14, 2021 and steers began grazing on December 9, 2021.

Daily mean temperature and precipitation (NOAA) during the growing period and grazing season of experiment 1 are shown in Figure 1. Total precipitation during the 74 d growing season (planting on August 12, 2020 to hard freeze on October 25, 2020) was 972 mm. There was 488 mm of precipitation with 363 mm as snowfall during the grazing season (November 12, 2020 to February 3, 2021). There were 36 d out of the 83 d of grazing with snow cover on the ground with an average depth of 9.1 cm. The average temperature during the grazing period was −1.2°C.

**Figure 2.**
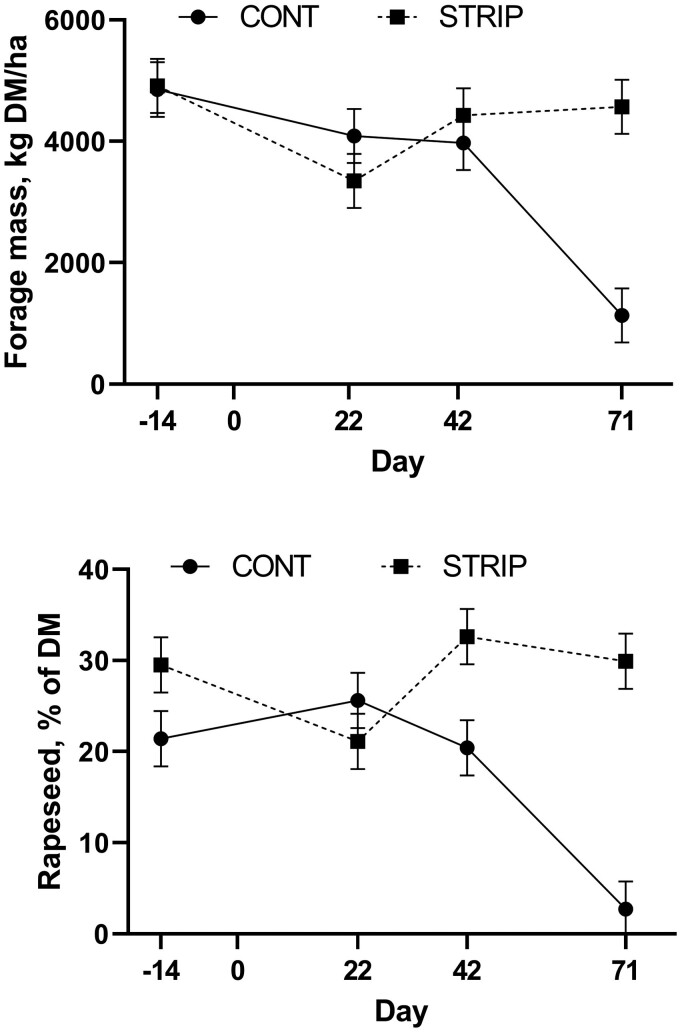
Total forage mass (DM basis) of oats (*Avena sativa*) and rapeseed (*Brassica napus*) mix and proportion of forage DM that was rapeseed in continuous grazed (CONT) and strip-grazed (STRIP) pasture in experiment 1. Grazing was initiated on day 1 (November 12, 2020) grazing ended on day 83. Treatment by day was significant (*P* = 0.01) for forage mass and proportion rapeseed.

There was a treatment-by-day effect (*P* < 0.01) for total forage mass and the proportion of forage that was rapeseed (Figure 2). Initial forage mass did not differ (*P* = 0.89) between treatments at 4,852 vs. 4,914 kg/ha for CONT and STRIP, respectively. The initial forage mass was predominantly oats with the rapeseed comprising 21% and 29% for CONT and STRIP, respectively which tended to differ (*P *= 0.08) between treatments. Additional forage mass samples were taken on grazing days 22, 42, and 71. These were taken within the whole CONT paddock and ungrazed area of STRIP to compare the forage that would be offered. After 22 d of grazing, total biomass was not different (*P* = 0.12) between CONT (4,089 kg/ha) and the ungrazed area of STRIP (3,346 kg/ha). Additionally, the rapeseed proportion did not differ (*P *= 0.31) between treatments at 26% and 21% for CONT and ungrazed STRIP, respectively. Forage mass on day 42 of grazing was still not different (*P *= 0.33) between treatments with 3,972 and 4,426 kg/ha for CONT and STRIP, respectively. However, CONT pastures had less (*P *= 0.01) rapeseed at 20% compared to STRIP pastures which had 32%. On day 71, forage mass was less (*P* < 0.01) for CONT (1,133 kg/ha) vs. STRIP (4,566 kg/ha) which is not surprising as forage mass in the STRIP treatment was sampled from the ungrazed portion of the field and therefore had no grazing pressure. On day 71, there was an even larger difference (*P *< 0.01) for rapeseed proportions between treatments, with 2.7% of the forage in CONT pastures being rapeseed compared to 30% in STRIP pastures. This is likely due to the selectivity of the cattle when grazing in the CONT. When comparing the relative proportion of forage mass attributed to rapeseed within treatments there was less (*P *< 0.01) rapeseed after 71 d of grazing for the CONT treatment compared to initial values; however, there was no difference (*P *= 0.92) for the STRIP treatment.

**Figure 3.**
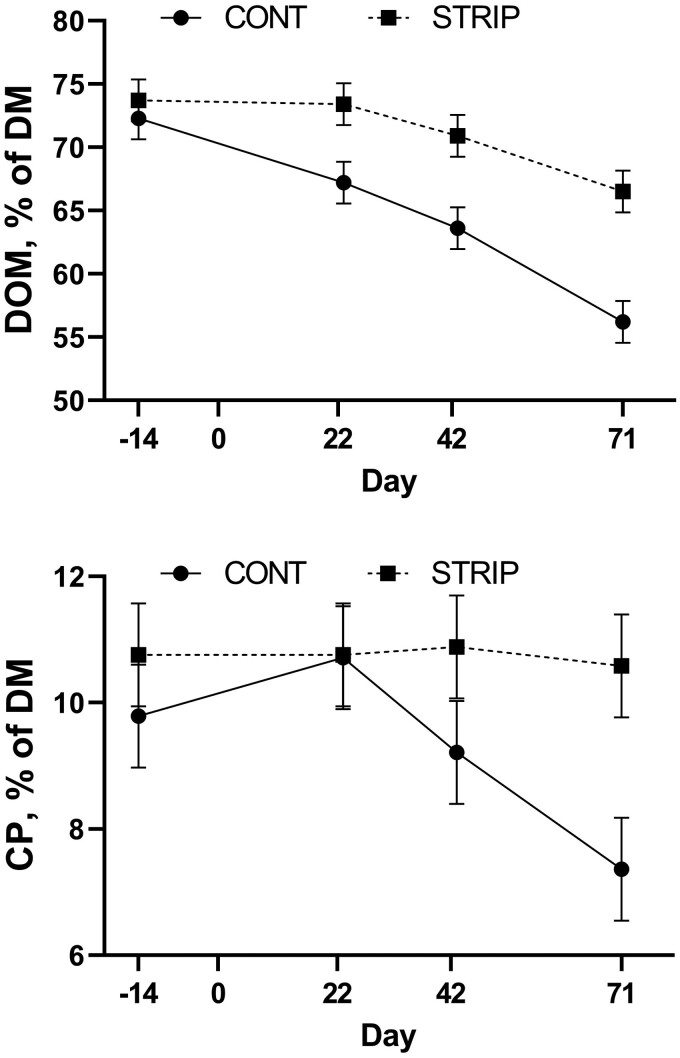
Digestible organic matter (DOM) and crude protein (CP) content of oats (*Avena sativa*) and rapeseed (*Brassica napus*) forage mix in continuous grazed (CONT) and strip-grazed (STRIP) pasture in experiment 1. Grazing was initiated on day 1 (November 12, 2020) and ended on day 83. Treatment by day tended (*P* = 0.07) to be significant for DOM and was significant (*P* = 0.05) for CP.

Initially, the forage on offer was relatively high in energy (73% DOM) and there was no difference (*P* = 0.58) between treatments (Figure 3). However, by day 22 of grazing, forage DOM was less (*P *= 0.02) in CONT pastures than in the ungrazed area of STRIP. This difference (*P *≤ 0.02) in the energy (DOM) content of the forage on offer continued throughout the rest of the grazing period. By day 71, there was an 18% decrease (*P *< 0.01) in the energy (DOM) content of CONT pastures compared to STRIP.

When evaluating the change in energy content of the forage over time within each treatment, CONT had a significant decrease (*P *≤ 0.03) in energy content across each of the sampling time point with initial energy content measuring 22% greater compared to energy content after 71 d. Alternatively, the energy content of STRIP forage was not different (*P* ≥ 0.21) from initial on either day 22 or 42, but by day 71 of grazing STRIP pastures were 10% lesser (*P *< 0.01) energy compared to initial. For within treatment variation across time, it is important to note that changes in STRIP treatments are a result of weathering (impacts of freeze/thaw cycles and precipitation) as forage samples were collected prior to grazing the strip. Alternatively, differences observed over time within the CONT treatment were a result of the combination of weathering, cattle selectivity, and trampling.

CP was not different (*P* ≥ 0.17) between treatments prior to grazing initiation, at day 22, as well as day 42 of grazing (Figure 3). However, by day 71 of grazing, CONT pasture contained an average of 3.2 ± 1.2 less (*P* = 0.02) percentage units of CP compared with forage in the ungrazed area of STRIP. Furthermore, within treatment, the CP content of CONT pastures did not differ through day 42 of grazing, but on day 71, CP content was significantly less (*P* ≤ 0.03) compared with CONT at all other time points. Alternatively, the CP content of the ungrazed area of STRIP pastures did not differ (*P *≥ 0.69) over time. This suggests that the difference between CONT and STRIP on day 71 was likely due to selectivity when grazing.

**Figure 4.**
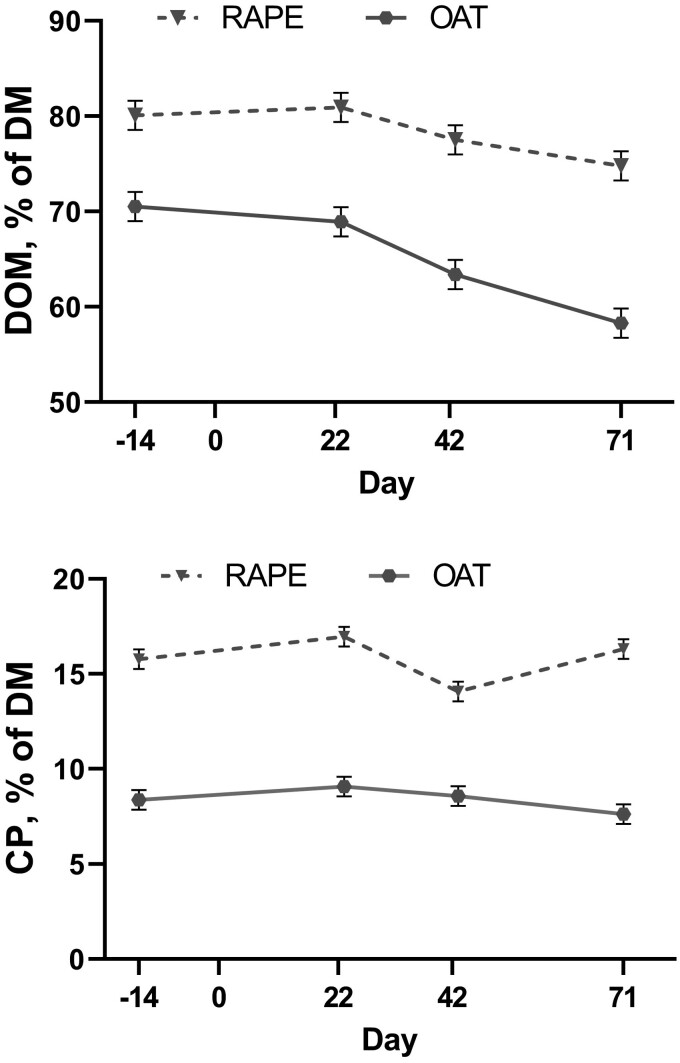
Digestible organic matter (DOM) and crude protein content of oats (OAT) and rapeseed (RAPE) across grazing season in experiment 1. Grazing was initiated on day 1 (November 12, 2020) and ended on day 83. Species by day tended (*P* = 0.06) to be significant for DOM and was significant (*P* < 0.01) for CP.

When evaluating the nutritive content of the 2 forage species within experiment 1, rapeseed contained greater (*P *< 0.01) DOM and CP when compared with oats at each sampling time point (Figure 4). Rapeseed DOM was not different (*P* ≥ 0.15) from initial through 42 d of grazing; however, by day 71, rapeseed was 5.5% units lesser (*P *< 0.01) than day −14. The DOM content of rapeseed on day 22 tended to be greater (*P *= 0.06) than day 42 and was significantly greater than day 71. The energy content of rapeseed on day 42 was not different (*P* ≥ 0.14) from day 71. The initial CP content of the rapeseed was lesser (*P *= 0.05) than after 22 d of grazing (Figure 4). However, the initial CP was greater (*P *< 0.01) than after 42 d of grazing and not different (*P *= 0.37) from 71 d of grazing. Rapeseed CP content after 42 d of grazing was less (*P *< 0.01) than either 22 or 71 d of grazing which were not different (*P *= 0.26) from each other. Overall, the CP content of rapeseed remained high with the lowest value being 14.0 ± 0.5% CP.

For oat, early in the grazing season, initial and day 22, DOM was not different (*P *= 0.36) from each other and were greater (*P *< 0.01) compared to DOM content later in the grazing season (days 42 and 71) which were not different (*P *= 0.14) from each other. When evaluating CP, the initial CP of oats was not different (*P* ≥ 0.18) from samples taken throughout the grazing season. After 22 d of grazing oat CP content was 1.5 ± 0.6% greater (*P *= 0.01) than after 42 d. After 42 d of grazing, CP content tended (*P *= 0.10) to be 1.0 ± 0.6% greater than after 71 grazing days.

Though disappearance could not be calculated due to the lack of a final forage mass collection, a possible decrease in forage lost to trampling by the STRIP treatment can be observed by the significant increase in the AUM per hectare able to be supported (*P* = 0.03) compared to the CONT (Table 2).

**Table 2.** Carrying capacity and performance of steers grazing an oat-rapeseed mix continuously (CONT) or strip-grazed (STRIP) over an 83-d period in the fall/winter in Eastern Nebraska (experiment 1)

Variable	CONT	STRIP	SEM	*P*-value
Initial BW, kg	238	238	0.3	0.54
Final BW, kg	312	300	1.3	0.01
ADG, kg	0.90	0.76	0.012	0.01
AUM/ha^1^	3.69	6.70	0.385	0.03
Gain, kg/ha	166	260	10.4	0.02
Cost of gain, $/kg	1.51	1.24	0.062	0.09

^1^AUM, animal unit month, a 454-kg animal over a month of time.

The initial BW of steers did not differ (*P *= 0.54) between treatments. Following grazing termination, STRIP steers were significantly lighter (*P *= 0.01) than CONT (Table 2). This is because STRIP steers had less (*P* < 0.01) ADG (difference of 0.14 kg/d) compared to CONT steers. However, due to the earlier described reduction in land grazed, STRIP steers gained 56% more kilogram per hectare (*P* ≤ 0.01) than CONT steers.

Despite the additional $69/ha associated with the labor of moving fence allocations twice weekly (Table 1), differences were found in favor of strip grazing in the partial budget economic analysis. Strip grazing tended (*P* = 0.09) to reduce the cost of gain by 18% compared to continuous grazing (Table 2).

### Experiment 2—Eastern Nebraska Year 2: Diverse Annual Mix

Daily mean temperature and precipitation during the growing period and grazing season of experiment 2 are shown in Figure 1. The total precipitation during the 99-d growing season (planting on July 14, 2021 to hard freeze on October 22, 2021) was 2,962 mm. There was 246 mm of precipitation with 89 mm as snowfall during the grazing season (December 9, 2021 to February 1, 2022). There were 5 d out of the 54 d of grazing with snow cover on the ground with an average depth of 2.5 cm. The average temperature during the grazing period was −3.9°C.

The initial total forage mass was not different (*P* = 0.44) between treatments (Table 3). Additionally, plant functional groups (grasses, forbs, legumes, seedheads, and sunflower heads) were compared between treatments. Grasses were not different between treatments (*P *= 0.56) and accounted for the majority (73%) of the forage mass. Visually the grasses appeared to be predominantly pearl, German, and browntop millet. Seedheads were predominantly from German and browntop millet and were not different (*P *= 0.81) between treatments. Forbs were mostly sunflower stems with a small amount of buckwheat and accounted for 9.3 ± 1.8% in CONT vs. 8.0 ± 1.8 for STRIP which was not statistically different (*P *= 0.66). The proportion of sunflower heads (1.9% of forage mass) was also not statistically different (*P* = 0.74) between treatments. Legumes were mostly cowpea, mungbean, spring pea, and vetch and were not different between treatments (*P* = 0.42) accounting for 4.7 ± 0.5% and 4.0 ± 0.5% for CONT and STRIP, respectively.

**Table 3.** Forage mass and disappearance of summer-planted 17-species mix when continuously grazed (**CONT**) or strip-grazed (**STRIP**) in the fall/winter in Eastern NE (experiment 2)

Variable	CONT	STRIP	SEM	*P*-value
Initial mass, kg/ha	2813	2487	238.6	0.44
Final mass, kg/ha	1521	1533	57.5	0.90
Disappearance, kg DM/AUM^1^	436	237	94.1	0.27
Initial forage mass, % of DM				
Grasses	71.3	73.7	2.39	0.56
Grass seedheads	12.3	12.7	0.84	0.81
Forbs and legumes	14.0	12.0	2.27	0.60
Sunflower heads	1.7	2.0	0.62	0.74
Disappearance, % change from initial forage mass				
Grasses	36.0	29.3	10.2	0.69
Grass seedheads	81.0	74.7	3.2	0.29
Forbs and legumes^2^	54.7	27.7	15.0	0.33
Sunflower heads	100	100	—	—

^1^AUM, animal unit month = 454-kg animal grazing over a month of time; calculated based on the weight and number of the grazing animals and duration of grazing; expected intake would be 318 kg of DM per AUM.

^2^Mostly sunflower stems.

Due to the small amounts of some functional groups, treatment replicates were combined prior to nutrient analysis (Table 4). Altogether, the total forage offered was 54% DOM and 6.9% CP. When broken into individual functional groups, the grasses (minus seedheads) had relatively low nutritive value with an energy content of 53% DOM and 5.7% CP. While grass seedheads had fairly high nutritive content at 65% DOM and 9.9% CP. German and browntop millet seedheads contained 32 and 20% starch (DM basis), respectively. Forbs (sunflower stems and buckwheat) were very low in nutritive content at 46% DOM and 6.8% CP, although the separated sunflower heads which were a relatively small fraction of the forage mass were high in nutritive content at 64% DOM and 10.9% CP. It should also be noted that these sunflower heads had started to fill with seed and had 7.5% fat on a DM basis. Similarly, although legumes were a small proportion of the forage on offer, they also had a relatively high nutritive value with 66% DOM and 17.1% CP.

**Table 4.** Initial energy (DOM) and protein (CP) content of forage components of 17-species mix grazed in the winter in Eastern Nebraska (experiment 2)

Forage type	DOM^1^, %	CP^2^, %
Grasses^3^	52.5	5.7
Grass seedheads^4^	65.1	9.9
Legumes^5^	66.1	17.1
Forbs^6^	45.6	6.8
Sunflower heads	63.9	10.9
Forage as offered	54.2	6.9

^1^DOM, digestible organic matter.

^2^CP, crude protein.

^3^Mostly pearl, German, and browntop millet.

^4^German and browntop millet.

^5^Cowpea, mungbean, spring pea, and vetch.

^6^Mostly sunflower stems with some buckwheat.

Post-graze forage mass was also not different (*P* = 0.90) between treatments (Table 3). The amount of grasses remaining in post-grazing was not different (*P *= 0.56) and accounted for 71.3 ± 2.4% in CONT pastures compared to 73.8 ± 2.4% in the STRIP pastures. Grass seedheads were also not different (*P* = 0.75) between treatments accounting for 4.0 ± 1.3% for CONT and 4.7 ± 1.3% for STRIP. For the final forage mass collection, the functional group of legumes had very little mass and the little remaining amount was combined into the forbs functional group. The percentage of forbs/legumes remaining was not different (*P* = 0.71) between treatments accounting for 11.7% and 13.3% for CONT and STRIP pastures, respectively. Sunflower heads were completely utilized and none remained in either treatment. Steers were allowed to be selective and as such utilization of the various functional groups was not different (*P* ≥ 0.29) between treatments (Table 3). Total disappearance in kilogram of DM per AUM was not different (*P* = 0.27) between the CONT and STRIP (Table 3).

Animal performance is displayed in Table 5. As designed, the initial BW was not different (*P* = 0.71) between the CONT and STRIP treatments. Following the 54-d grazing season, neither final BW nor ADG differed (*P* ≥ 0.56) between treatments. However, there was a tendency (*P = *0.10) for a 43% increase in carrying capacity. Furthermore, gain per hectare was increased (*P = *0.02) as STRIP steers gained an additional 29 kg/ha more than CONT steers. Even so, the cost of gain was not different (*P *= 0.34) between treatments, suggesting that the increase in labor balanced with the increase in gain per hectare.

**Table 5.** Carrying capacity and performance of steers grazing a summer-planted 17-species mix continuously (CONT) or strip-grazed (**STRIP**) over a 54-d period in the fall/winter in Eastern Nebraska (experiment 2)

Variable	CONT	STRIP	SEM	*P*-value
Initial BW, kg	288	288	0.2	0.71
Final BW, kg	326	323	2.4	0.57
ADG, kg	0.70	0.66	0.04	0.56
AUM/ha^1^	2.95	4.21	0.31	0.10
Gain, kg/ha	93	122	3.3	0.02
Cost of gain, $/kg	2.02	1.91	0.06	0.34

^1^AUM, animal unit month, a 454-kg animal grazing for one month; calculated based on the weight and number of the grazing animals and duration of grazing.

## DISCUSSION

Following the harvest of small cereal grains, there is an opportunity to capitalize on the remaining growing degree days by cultivating annual forage crops. The characteristics and performance of annual forage species are influenced by a multitude of factors, as highlighted in previous research (Baron et al., 1999; Keogh et al., 2011; Landry et al., 2019). Factors such as geographical location, environmental conditions, time of use, and planting date all play pivotal roles in shaping the growth, quality, and suitability of various annual forage species.

Strip grazing has been previously shown to increase forage utilization in perennial forages compared to continuous grazing (Blount et al., 1991). Pasture rental rates have increased by 76% over the last 2 decades (USDA NASS, 2023) and hay prices have increased by 40%, the rising cost of forage accentuates the importance of enhancing grazing efficiency. However, this increase in productivity comes at the cost of increased labor. With labor shortages plaguing many producers, it becomes important to understand the effects of strip grazing stockpiled annual forages.

Over 2 winter grazing seasons, 2 different annual forage systems (oats and brassicas or diverse species mixture) were either strip-grazed or continuously grazed to compare steer performance and forage utilization. Interestingly, the magnitude of animal response to strip grazing was different between the 2 experiments. In both experiments, the amount of gain per hectare was increased when strip grazing was utilized compared to continuous grazing. However, in experiment 1, STRIP steers gained an additional 56% per hectare compared to their CONT counterparts whereas in experiment 2 STRIP steers only gained an additional 31%.

Though gain per hectare was not analyzed in their study, Blount and et al. (1991) found that strip grazing allowed for the same total gain on less hectares of land when grazing actively growing native flood meadows containing predominantly a mix of meadow foxtail (*Alopecurus pratensis*), saltgrass (*Distichlis stricta*), reed canarygrass (*Phalaris arudinacea*), quackgrass (*Agropron repens*), Neveda bluegrass (*Poa nevadensis*), sedges (*Carex* spp.) and rushes (*Juncus*, spp.). By utilizing strip grazing on stockpiled forages, producers may be able to achieve an overall higher level of animal gain on their current land base. In the current study, strip grazing resulted in an 82% increase in the carrying capacity of the oat/brassica mixture and tended to increase the carrying capacity of the diverse species mix by 43%. This increase in carrying capacity is the result of STRIP using 0.2 ha/steer less during the 83 d grazing period compared to CONT in experiment 1 and 0.11 ha/steer less during the 54-d grazing season in experiment 2. One of the premises of strip grazing is that it reduces loss due to trampling compared with continuous grazing, and this results in increased carry capacity. Therefore, strip grazing can stretch feed resources by allowing more animals grazing days on the same land base which can reduce dependence on stored forages.

In the current experiments, the same post-graze mass was targeted between treatments using visual estimates. Unfortunately, post-graze measurements were not taken in experiment 1, while visually the amount of forage at the end of grazing was similar between treatments, it is possible that more of the forage mass disappeared in the STRIP than CONT, causing the increase in carrying capacity. However, in experiment 2, post-graze mass, determined by clipping the forage, did not differ between treatments, suggesting that strip grazing did indeed result in improved utilization, likely through less tramping losses.

Weather may play a role in the impact of strip grazing vs. continuous grazing. For instance, precipitation, especially in the form of rain could result in more trampling losses, increasing the carrying capacity benefit of strip grazing. Experiment 1 had twice as much total precipitation (rain plus snow) as experiment 2. There were 6 d in the 83 d grazing season (7% of days) in which above freezing temperatures and rainfall occurred, while in experiment 2, there were only 2 d in the 54-d grazing season (4% of days).

Even though animal productivity per unit of land increased, it is commonly accepted that it comes at the expense of individual animal performance. It would be expected that initially continuously grazed cattle walk and select the most palatable, and potentially nutritious, components of the forage first and trample forage in the process whereas strip-grazed cattle are forced to eat the forage more uniformly over time. Individual cattle performance measured by ADG was reduced by 16% in experiment 1 and did not differ in experiment 2 though there was a numerical reduction of 6%. There are many possible explanations for the observed differences in animal performance between the 2 experiments. The nutritive value of the forage differed between the 2 experiments. When steers are offered higher-quality forages, they are expected to perform better compared to when they are offered lower-quality forages. In experiment 1, a high-quality oat and brassica mixture (73% DOM and 10.3% CP) was utilized. In experiment 2, a 17-species mix was used, which was dominated by growth of the warm-season millets, resulting in the forage on offer being lower energy and protein (54% DOM and 6.9% CP). It is possible that the measured differences in individual animal performance response and magnitude of response in gain per hectare could have been a result of differences in forage quality.

Another possible explanation for the difference observed between the 2 experiments is the amount of forage allocated. When cattle are allocated more forage, their DM intakes increase in a curvilinear fashion (Greenhalgh et al., 1966). In experiment 1, the continuously grazed treatments had an average of 1,245 kg DM offered/AUM while those strip grazing had an average of 719 kg DM offered/AUM. In experiment 2, CONT treatments had 894 kg DM offered/AUM while STRIP treatments had 631 kg DM offered/AUM. The amount of forage allocated for STRIP grazing was numerically similar between years. However, there was a 50% reduction in the amount of forage allocated to STRIP compared to CONT for experiment 1 and only a 29% reduction in forage allocation in experiment 2. Again, the goal was to have the post-graze mass at the end of the grazing period be similar between treatments, with any differences in the amount of forage offered being due to differences in trampling losses. Previous literature has highlighted that the performance of animals when strip grazing is correlated with allocation amount. When strip grazing, Curtis et al. (2008) noted that decreasing the allocation amount of stockpiled tall fescue as a percentage of BW for lactating cows resulted in a linear decrease in nursing calf ADG.

Lehman et al. (2021) suggested that continuously grazing winter resources results in a decreasing availability of high-quality nutrients as the grazing season continues due to the selective nature of grazing cattle. They suggested that utilizing strip grazing could provide a more stable plane of nutrition over the grazing period (Lehman et al., 2021). In experiment 1, there was a decrease in rapeseed proportions from grazing initiation to 71 d of grazing in the CONT treatment suggesting that steers in experiment 1 were selecting the higher-quality component of the forage earlier in the grazing season. Since steers managed under strip grazing were receiving a new portion of the field twice weekly, they would not be able to graze as selectively as CONT. Instead, steers in STRIP would have had to consume a higher proportion of oats considering they had access to approximately half the kilogram of forage mass per AUM. This could be the reason we saw a difference in individual animal performance in experiment 1. In experiment 2, there appeared to be no difference in utilization of plant functional groups and thus no difference in selectivity between CONT and STRIP. The combination of lack of difference in selectivity and lesser forage offered for CONT in experiment 2, compared to experiment 1, could be the reason there was no difference in the individual animal performance in experiment 2. Additional research is needed to determine how strip grazing affects the utilization of forage mixtures and therefore the plane of nutrition and individual animal performance.

Strip grazing is more expensive per hectare due to the increased labor required compared to continuous grazing. The additional cost of labor from moving fences has been previously observed for other management-intensive grazing systems (Walton et al., 1981). Labor costs will vary depending on the travel distance required and the size of the group being moved. However, it is important to understand if the increase in carrying capacity and gain per hectare is enough to offset the additional cost of labor. If we take the costs that were consistent between treatments ($250.42 and $187.80/ha in experiments 1 and 2, respectively) and divide them by the steer grazing d/ha achieved, the costs for extra labor and fence associated with strip grazing could equate to $0.63 and $0.62 steer/d in experiments 1 and 2, respectively, to have the same cost per day as continuous. If we do a similar calculation on a gain basis, an additional $0.40 or $0.39 steer/d in experiments 1 and 2, respectively, could be charged for extra labor and fence. This suggests that the additional gain from the increase in harvest efficiency can pay for the additional labor in many situations. However, it is important to point out that strip grazing might not always pay. When strip grazing actively growing pastures through the spring and summer months, Blount et al. (1991) saw a decrease in ADG with and similar total gain on less hectares of land. The decrease in ADG is likely due to an observed decrease in diet quality selected by steers when grazing strip treatment pastures compared to continuous treatment. This difference persisted for roughly 42 d of the grazing season and was speculated by the authors to be a result of advanced forage maturity in the STRIP treatments compared to the CONT treatment where steers were better able to graze regrowth. This suggests that strip grazing may be most beneficial with stockpiled dormant forages rather than in-season grazing.

In the current study, the experimental unit was pasture and only 3 replicates were used, potentially contributing to the observed lack of significance in some measures. Based on the results of this study and others, variability in individual animal response to strip grazing deserves further evaluation. Understanding how strip grazing affects cattle’s nutritional plane compared to continuous grazing requires additional research to assess changes in selectivity and nutrient availability throughout the grazing season in each system. As there are endless possibilities of summer-planted annual forages available for strip grazing during the winter, future research should explore the potential interaction of species selection with strip grazing. Additionally, investigating how varying forage allocation and movement frequency influences animal performance in strip grazing systems is essential for optimizing the effectiveness of strip grazing.

## CONCLUSIONS

Access to affordable winter feed is a widespread challenge for cattle producers. Utilizing strip grazing for stockpiled annual forages can effectively boost carrying capacity and gain per hectare during fall and winter. However, the impact on individual animal performance varies. Disparities in response to strip grazing could stem from differences in forage quality, allocation and weather. Further research is needed to refine and optimize the utilization of this management approach.
